# FKBP51 protects 661w cell culture from staurosporine-induced apoptosis

**Published:** 2011-05-04

**Authors:** Donald Raymond Daudt, Thomas Yorio

**Affiliations:** Department of Pharmacology and Neuroscience University of North Texas Health Science Center Fort Worth, TX

## Abstract

**Purpose:**

Neurodegenerative diseases and neurotraumas typically result in apoptosis of specific neurons leading to the pathology observed during the disease state. Existing treatments target the symptoms instead of preventing the death of these neurons. Although neuroprotective drugs should be useful as a treatment to prevent further loss of neurons, efficacious molecules are lacking. FK506 (tacrolimus), a widely used immunosuppressant drug, has significant neuroprotective and neuroregenerative properties throughout the central nervous system, including the eye. FK506 achieves these properties through interaction with FK506 binding proteins (FKBP), including FK506 binding protein 51 (FKBP51). In this study, we examine the effects of FKBP51 as a neuroprotective agent on a neuronal cell line.

**Methods:**

We cultured 661w cell cultures with or without FK506, or stably transfected them with an FKBP51 expression vector. These cells were then exposed to the apoptosis-inducing agent staurosporine. Cell viability was determined using a calcein AM/propidium iodide assay. Protein levels and activation of nuclear factor kappa-light-chain-enhancer of activated B cells (NF-κB) were determined by western immunoblot analysis.

**Results:**

FKBP51 overexpression significantly protected 661w cell cultures from staurosporine-induced apoptosis. FKBP51 overexpression also significantly increased NF-κB p65 protein levels and activated NF-κB p65. FK506 treatment significantly protected 661w neuronal cultures from staurosporine-induced apoptosis. FK506 increased FKBP51, NF-κB p65, and levels of activated NF-κB p65 protein.

**Conclusions:**

These results suggest that FKBP51 protects 661w cell cultures from apoptosis induced by staurosporine. Additionally, FK506 protected 661w cell cultures from apoptosis and displayed a mechanism similar to that of FKBP51 overexpression. Both FK506 and FKBP51 appear to act through activation of NF-κB p65 protein, suggesting a common pathway for neuroprotection. These findings implicate FKBP51 as a protein important to neuronal cell culture survival. FKBP51 may be a potential therapeutic drug target for preventing the neurodegeneration and neurotrauma that occur during neurodegenerative diseases.

## Introduction

Neurodegenerative diseases such as Alzheimer disease, Parkinson disease, and glaucoma affect the lives of millions and are increasing in prevalence due to the progressive increase in human lifespan [1]. Each year, over 3 million people worldwide die from neurologic disorders such as Alzheimer and Parkinson disease [2]. Typical treatments for neurologic disorders specifically target treating the symptoms of each individual disease and are not directed to intervening in the disease process. However, it is known that apoptosis accounts for most neuronal cell death during neurologic disorders [3]. This similarity provides hope that neuroprotectant intervention could be uniformly beneficial to several neurodegenerative disorders; however, efficacious neuroprotectants are currently unavailable [4].

FK506 (tacrolimus) exhibits significant neuroprotective and neuroregenerative properties in several forms of neurotrauma, including optic nerve crush, traumatic brain injury, brain ischemia, sciatic nerve injury, and focal and global ischemia [5-8]. This protection is not limited to neurons; it extends to glia cells within the brain and several other organs [9]. These characteristics of FK506 make it potentially useful for neuroprotection; however, FK506 produces calcineurin-induced immunosuppression by binding FK506 Binding Protein 12 (FKBP12), which can increase the incidence of cancer [10,11]. FK506 was found to be equipotent in protecting cells lacking FKBP12 (U251 human glioma), compared to cells expressing FKBP12 (SH-SY5Y human neuroblastoma) [12]. Furthermore, FK506 also was found to protect neurons in FKBP12 knockout mice [13]. FK506 drug analogs, such as GPI-1046, which function independently of FKBP12, were shown to be neuroprotective [12,14]. However, not all FK506 downstream signaling pathways have been defined. FK506 interacts through several binding proteins, leading to several neuroprotective and neuroregenerative traits devoid of calcineurin inhibition [15]. Characterization of these signaling pathways would be advantageous to treating neurodegenerative diseases without systemic immunosuppression.

FKBP51, an immunophilin that interacts with FK506, is a potential neuroprotective agent for preventing apoptosis during neurodegenerative disease and neurotrauma. FKBP51 plays a significant role in the activation of nuclear factor kappa-light-chain-enhancer of activated B cells (NF-κB), an important cell-survival protein. The activation of NF-κB is initiated through the degradation of the the inhibitory molecule, IKappaB (IκB). This leads to the activation and translocation of NF-κB into the nucleus to initiate transcription of several prosurvival proteins, growth factors, and anti-apoptotic proteins. IκB is ubiquitinated through the serine/thereonine kinase, IKappaB Kinase (IKK), leading to the degradation of IκB through a proteosome. FKBP51 is an important cofactor of the catalytic subunit (IKKα) of IKK [16]. Overexpression of FKBP51 has been shown to upregulate NF-κB protein levels in hematopoietic cells [17]. This suggests a new potential neuroprotective and regenerative mechanism of FKBP51 [18]. Furthermore, NF-κB regulates the transcription of several anti-apoptotic proteins, including BCL-2 [19]. In a melanoma cell line, siRNA-mediated reduction of FKBP51 protein levels decreased expression of NF-κB and increased IκBα and IκBβ protein levels [20]. In UT7 cells, FKBP51 overexpression increased the protein levels of NF-κB p65 and NF-κB p50, and decreased the protein levels of IκBα [17]. Sustained activation of NF-κB was neuroprotective against glutamate-induced excitotoxicity in primary cortical neurons [21].

FKBP51 is a potential neuroprotective target; however, it is unclear if FKBP51 plays a neuroprotective role. Currently, we are testing the hypothesis that increases in FKBP51 protein levels decrease 661w neuronal cell culture death in reaction to the apoptosis-inducing agent staurosporine [22-24]

## Methods

### 661w cell culture

The 661w cells were derived from a murine retinal tumor (these have been shown to have the same relevant cellular and biochemical characteristics of cone photoreceptor neurons [25]). The 661w cells were grown in Dulbecco’s modified Eagle’s medium (DMEM, cat no. 23700–040; Invitrogen-Gibco, Grand Island, NY), supplemented with 10% heat-inactivated fetal bovine serum (cat no. 26140–079; Invitrogen-Gibco), 100 U/ml penicillin, and 100 mg/ml streptomycin (Fisher Scientific, Pittsburgh, PA). The 661w cells were cultured at 37 °C in 5% CO_2_ and air for all experiments.

### Stable transfection

The pCL-neo-FKBP51 overexpression vector and parental control pCl-neo vector were a kind gift from Dr. Marc B. Cox, University of Texas El Paso, El Paso, TX. Vectors were reconstituted in sterile Tris/EDTA buffer and transformed into DH5α *E. coli*. Competent bacteria were selected using ampicillin LB plates. Colonies that produced the highest levels of vectors were selected through miniprep. Maxipreps were performed using a CsCl gradient. Vectors were rehydrated in sterile TE buffer and maintained at −20 °C.

The 661w neuronal cell cultures (passage 6) were seeded in 100 mm dishes and grown with complete DMEM. After 24 h, the cell cultures were transfected through a lipophilic method (Metafectene Pro; Biontex, Toulouse, France), as instructed by the manufacturer. After an additional 24 h, 2 mg/ml of G418 were added to kill cells that did not incorporate the vectors. Cells were maintained on 0.2 mg/ml G418 and grown to a maximum 15 passages.

### Western blot analysis

Cultured cells, from cells grown at 60%–80% confluence, were isolated and lysed as previously described [26]. Protease inhibitors (1 mM Dithiothreitol [DTT] and 500 µM Phenylmethanesulfonyl fluoride [PMSF]) were added. Cells were harvested at 60%–80% confluence. Protein concentrations were determined using Bio-Rad Bradford Protein Assay (500–0006; Bio-RaD, Bio-Rad Laboratories, Hercules, CA). Samples of protein (25 μg) were run on a sodium dodecyl sulfate 7.5% polyacrylamide gel and immunoblotted according to previous published methods [27-29]. Briefly, the separated protein was transferred to 0.45 μm-supported nitrocellulose membranes (162–0094; BioRad) and blocked with 7.5% nonfat dry milk in Tris-buffered saline with Tween. The following primary antibodies were used: mouse anti-FKBP51 (610582, 1:500; BD Transduction Laboratories, Lexington, KY); mouse anti-NF-κB (SC-8008, 1:500; Santa Cruz, Santa Cruz, CA); mouse anti-GAPDH (P04406, 1:1000; Millipore, Billerica, MA); rabbit anti-phospho-NF-κB (3033, 1:500; Cell Signaling, Cell Signaling Technology, Inc., Danvers, MA); and mouse anti-β-tubulin (T0198, 1:1,000; Sigma, Sigma-Aldrich, St. Louis, MO). Primary antibodies were incubated and rotated overnight at 4 °C. Blots were washed for 30 min at room temperature. Prior to the addition of secondary antibodies ECL-rabbit IgG, HRP-linked, or ECL-mouse IgG, HRP-linked (NA9340 and NA9310, 1:10,000; GE, Piscataway, NJ) for 30 min. Luminescence was detected using SuperSignal West Dura (34075; Thermo Scientific, Waltham, MA) in the BioRad Molecular Imager. Densitometric analysis was performed using the Bio-Rad Image Lab. GAPDH and β-tubulin were used as loading controls.

### Calcein-acetomethoxy/propidium iodide cell-survival assay

Cell viability was determined using a calcein/propidium iodide (cat. no. C3099; Invitrogen-Molecular Probes, Carlsbad, CA) dual-staining assay. The cell cultures were treated with or without 100 nM–10 μM staurosporine, an apoptosis-inducing agent [22-24], for 24 h (ALX-380–014-M001; Enzo Life Sciences, Plymouth Meeting, PA). After treatment, the culture medium was removed, and the coverslips were rinsed with 1× phosphate buffer saline (PBS). Then, 1 μM calcein and 2 µg/ml propidium iodide in 2 ml 1× PBS were added to each culture well. The culture dishes with the cells were incubated at 37 °C for 60 min, and fluorescence was measured (Microphot FXA digital fluorescent microscope; Nikon, Melville, NY).

### Caspase-3 detection assay

Caspase-3 activity was determined using the SR-DEVD-FMK Caspase-3 detection kit, Cell Technology Inc. (Mountain View, CA) following the manufacturer’s protocol. Coverslips were coated with 10 μg/ml Poly-D-Lysine for 60 min, washed, and then placed into wells with 500 μl of DMEM. Either 10,000 empty vector or FKBP51-overexpressing 661w cells were added to each well and incubated for 24 h. Staurosporine or a vehicle was added to each well to reach a final concentration of 10 nM, 100 nM, or 1 μM for 6 h. Cells were washed three times with PBS. Prepared caspase-3 detection reagent (300 μl) was added to each well and incubated for 60 min. Cells were washed three times with PBS and then inverted onto a slide with Fluorosave. Images were taken at the same exposure times on a fluorescence microscope (Microphot FXA digital fluorescent microscope; Nikon). Images were measured for intensity using Image J software.

### Statistical analysis

SigmaPlot 11.0 (Systat Software Inc., San Jose, CA) was used to perform all statistical analyses. Results were expressed as mean±standard error. Paired comparisons were analyzed using a Mann–Whitney U test. Multiple comparisons were performed using a one-way ANOVA (ANOVA) followed by a Mann–Whitney U test. Significance was defined as a p value of ≤0.05.

## Results

### FKBP51 expressed in rat retina and brain

**Figure 1:**
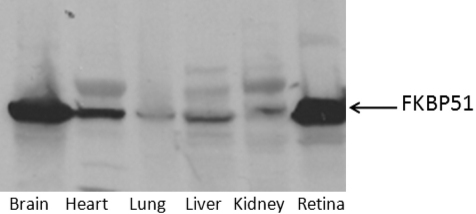
FK506 (tacrolimus) binding protein 51 is expressed in rat retina and brain. Tissues were extracted from an adult Sprague Dawley rat and the protein was isolated. Tissue lysates (50 μg) were added to each lane. Western blot analysis detected the protein in the brain, heart, lung, liver, kidney, and retina.

An adult Sprague Dawley rat was sacrificed and the brain, retina, heart, lung, liver, and kidney were collected in HEPES buffer containing protease inhibitors (1 mM DTT and 500 µM PMSF). Tissue and cells were lysed using sonication. Fifty micrograms of protein were added to each well. An anti-FKBP51 antibody (Cat: 610582; BD Transduction Laboratories) was used to detect FKBP51 protein. FKBP51 was detected in all tissues that were analyzed (Figure 1).

### FKBP51 overexpression increased protein levels of NF-κB and activated NF-κ and decreases protein levels of IκBα

**Figure 2:**
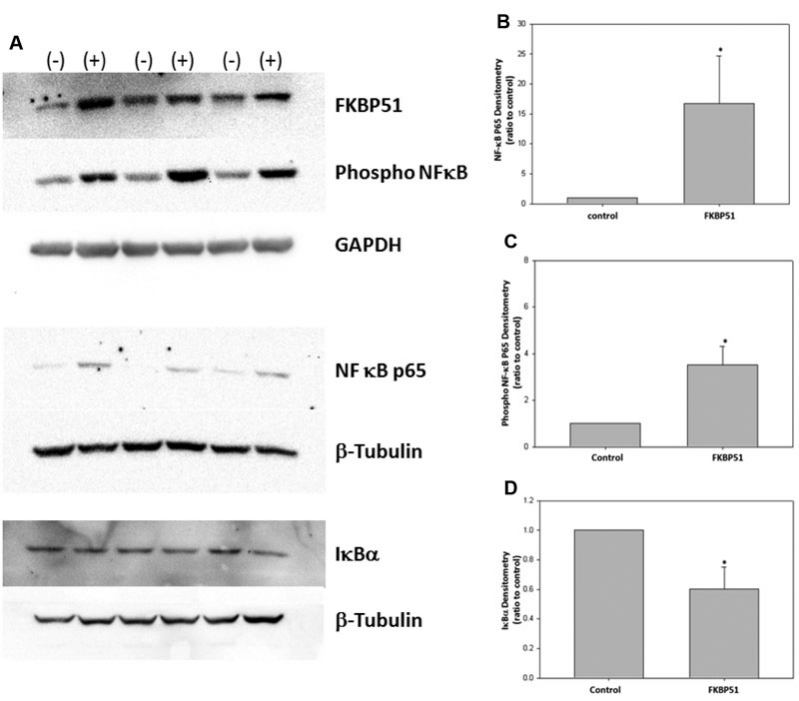
FK506 (tacrolimus) binding protein 51 overexpression increases protein levels of nuclear factor kappa-light-chain-enhancer of activated B cells (NF-κB), activated NF-κB, and decreased protein levels of inhibitory molecule, IKappaB (IκB). **A**: FK506 Binding Protein 51 (FKBP51) overexpression significantly increased NF-κB p65 and phospho NF-κB p65 protein levels while significantly decreasing IκBα protein levels. The 661w neuronal cell culture—stably transfected with an FKBP51 expression vector—increased FKBP51 protein levels fivefold, compared to the 661w neuronal cell culture stably transfected with the parental empty vector. (-) represents stably transfected empty vector control cells and (+) represents stably transfected FKBP51 overexpression cells. **B**, **C**: The increase in FKBP51 expression caused a significant increase in protein levels of NF-κB p65 and activated NF-κB p65 (NF-κB p65: n=6, *p=0.002; phospho NF-κB p65: n=6, *p=0.002). **D**: The increase in FKBP51 expression caused a significant decrease in protein levels of IκBα (IκBα: n=3, *p=0.044). The loading controls were β-tubulin for IκBα and NF-κB p65, while GAPDH was used for FKBP51 and for activated NF-κB p65. Significance was obtained through one-way ANOVA (ANOVA) and the Mann–Whitney test. Error bars represent SEM.

FKPB51 is expressed in many organs, including the retina (Figure 1). We used western blot analysis to determine the expression of FKBP51 and the potential downstream signaling molecules NF-κB p65 and phosphorylated NF-κB (n=6). FKBP51 was overexpressed fivefold in FKBP51 transfected cells, compared to cells transfected with the empty vector controls. The overexpression of FKBP51 also increased protein levels of phosphorylated NF-κB p65 by 3.2±0.5 fold and NF-κB p65 by 18±14 fold. β-tubulin (IκBα and NF-κB p65) or GAPDH (FKBP51 and phosphor NF-κB p65) was used for normalization (Figure 2).

### FKBP51 overexpression protected 661w cell culture from staurosporine

**Figure 3:**
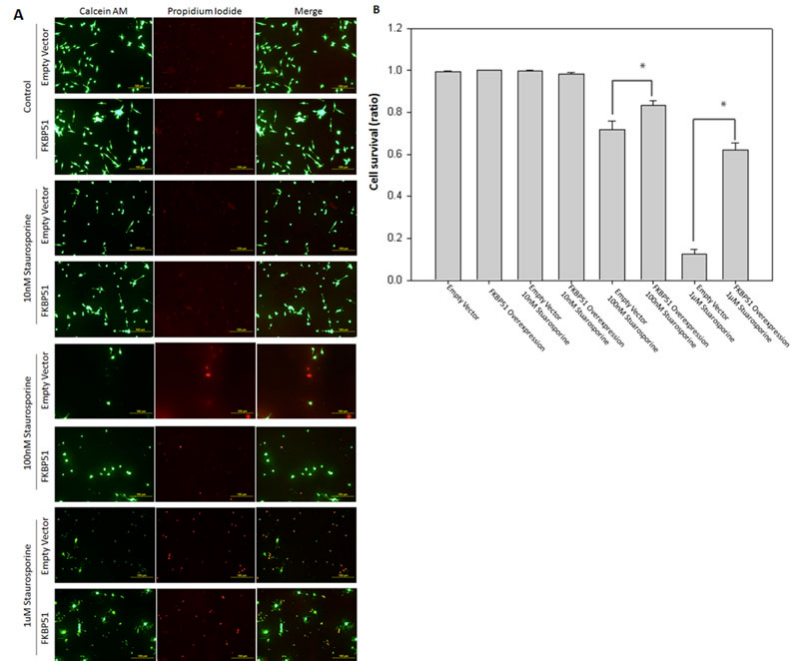
FK506 (tacrolimus) binding protein 51 overexpression protects 661w cell culture from staurosporine. **A**: The FK506 Binding Protein 51 (FKBP51) protein significantly protected the 661w neuronal cell culture from staurosporine-induced apoptosis. Cell survival was monitored with a calcein-AM/propidium iodide cell-survival assay. The 661w neuronal cell cultures were stably transfected with either an FKBP51 expression vector or parental empty vector. Cells were treated with or without varying concentrations of staurosporine (10 nM, 100 nM, and 1 µM) for 24 h (scale bar=100 µm). **B**: FKBP51 overexpression significantly protected the 661w neuronal cell culture from staurosporine-induced apoptosis at 100 nM (p=0.013) and 1 μM (p≤0.001) concentrations (n=3). Error bars represent SEM.

We wanted to determine whether an increase in FKBP51 protein would protect neuronal cell cultures from staurosporine, the apoptosis-inducing agent. Cell viability was determined using calcein AM/propidium iodide double-staining. In the absence of staurosporine, 99%–100% of the FKBP51 and empty-vector transfected cells remained viable. The addition of 10 nM staurosporine for 24 h did not induce a significant level of apoptosis in either cell culture. FKBP51 cells had 98±0.02% viability, and 99±0.01% of empty vector cells survived. In contrast, the addition of 100 nM staurosporine induced cell death in both FKBP51-overexpressing and empty-vector cell cultures. However, FKBP51 overexpression significantly protected the 661w neuronal cells from 100 nM staurosporine-induced apoptosis (83±0.02% viability in FKBP51 cells versus 71±0.04% viability in control cells, p=0.013). Furthermore, FKBP51 overexpression significantly protected the 661w neuronal cell culture from the 24 h of 1 μM staurosporine-induced apoptosis. The FKBP51 overexpression significantly protected the neuronal cells (62±0.03% viability in FKBP51 cells versus 12±0.02% viability in control cells, p≤0.001; Figure 3).

### FK506 treatment increased FKBP51 and NF-κB protein levels

Western blot analysis was used to determine the protein expression of FKBP51 and NF-κB p65 after 24 h of FK506 treatment (n=3). FK506 dose-dependently increased FKBP51 and NF-κB protein levels. FKBP51 was significantly elevated with 10 μM FK506 (p=0.023) and NF-κB at 100 nM (p=0.004).

**Figure 4:**
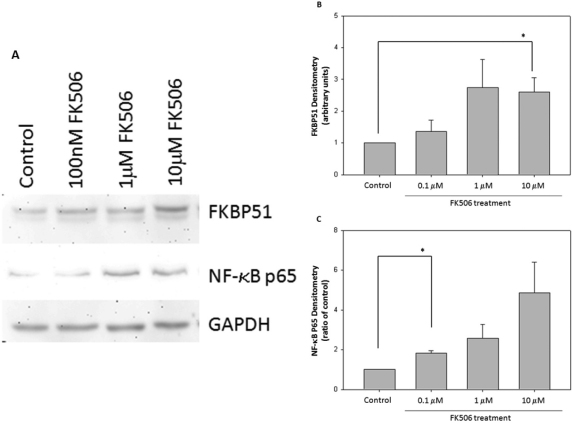
FK506 (tacrolimus) treatment increases FK506 binding protein 51 (FKBP51) and nuclear factor kappa-light-chain-enhancer of activated B (NF-kB) cell protein levels. **A**: Twenty-four hour FK506 treatments significantly increased protein levels of FKBP51 and NF-κB in the 661w neuronal cell cultures. The 661w neuronal cell cultures were treated for 24 h with a vehicle—100 nM FK506, 1 μM FK506, or 10 μM FK506. Cell lysate proteins (25 µg) were separated by sodium dodecyl sulfate PAGE (SDS–PAGE), transferred to membranes, and immunoblotted for FKBP51, NF-κB, and glyceraldehyde 3-phosphate dehydrogenase (GAPDH). The quantification of band intensity is represented as a percentage of FKBP51 or NF-κB of its corresponding GAPDH control band on the same membrane. **B**: FK506 significantly increased FKBP51 protein levels at a concentration of 10 μM (p=0.03), while 0.1 μM and 1 μM caused an increased in FKBP51 protein levels; however, significance was not obtained (n=3). Error bars represent SEM **C**: Additionally, FK506 significantly increased NF-κB protein levels at a concentration of 0.1 μM (p=0.004), while 1 μM and 10 μM caused larger increases in protein levels; however, significance was not obtained (n=3 for each dose indicated).

All three concentrations of FK506 (0.1 μM, 1 μM, and 10 μM) increased FKBP51 protein expression by 1.36±0.3 fold, 2.7±0.9 fold, and 2.6±0.4 fold, respectively. Furthermore, the same FK506 concentrations increased NF-κB p65 protein levels by 1.58±0.1 fold, 2.42±0.21 fold, and 5.6±0.4 fold, respectively. GAPDH was used as an equal loading control (Figure 4). Additionally, there were no changes to phosphorylated NF-κB p65 protein levels when analyzed during the 24 h treatment (unpublished data).

### One micromole FK506 treatment increased activation of NF-κB

**Figure 5:**
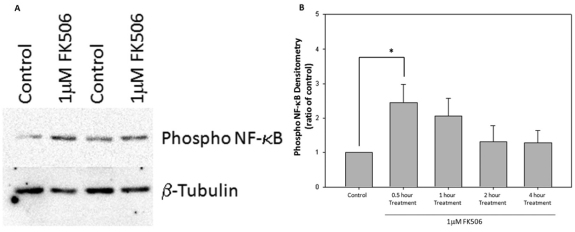
The 1 μM FK506 (tacrolimus) treatment increases activation of the nuclear factor kappa-light-chain-enhancer of activated B cells (NF-kB). **A**: The 1 μM FK506 treatment for 30 min significantly activated NF-κB protein in 661w neuronal cell cultures. These were treated with 1 μM FK506 or the control (DMSO) for 30 min, 1 h, 2 h, or 4 h. Cell lysate proteins (25 ug) were separated by western immunoblot analysis for phosphor-NF-κB and β-tubulin. The quantification of band intensity is represented as the percentage of phospho NF-κB of the corresponding control (β-tubulin) band on the same membrane. **B**: The 1 μM FK506 significantly increased phospho NF-κB protein levels (p=0.004) at 30 min, while phospho NF-κB protein levels returned to near basal levels within 2 h of FK506 treatment (n=6). Error bars represent SEM.

NF-κB is a nuclear transcription factor with diverse activities, including the regulation of cell survival [30]. The majority, but not all, of the available research indicates that NF-κB increases anti-apoptotic actions and prevents cell death in various cells [31-33]. It would be helpful to determine if FK506, like FKBP51, activates NF-κB, because it may be a common downstream molecule activated by both FK506 and FKBP51 to achieve neuroprotection. Western immunoblot analysis determined that the FK506 (1 μM) phosphorylates NF-κB p65 after 30 min, 1 h, 2 h, and and 4 h (n=6 at each time point). FK506 significantly increased the phosphorylation of NF-κB p65 2.2±0.5 fold (p=0.004) after 30 min. It was also determined that phosphorylated NF-κB p65 returned to baseline after 1 h, 2 h, and 4 h. β-tubulin was used as the equal-loading control. Therefore, the protein levels of NF-κB p65 increased after 24 h of treatment, while the phosphorylated form of NF-κB p65 increased sooner (Figure 5).

### FK506 protected 661w cell cultures from staurosporine-induced cell death

**Figure 6:**
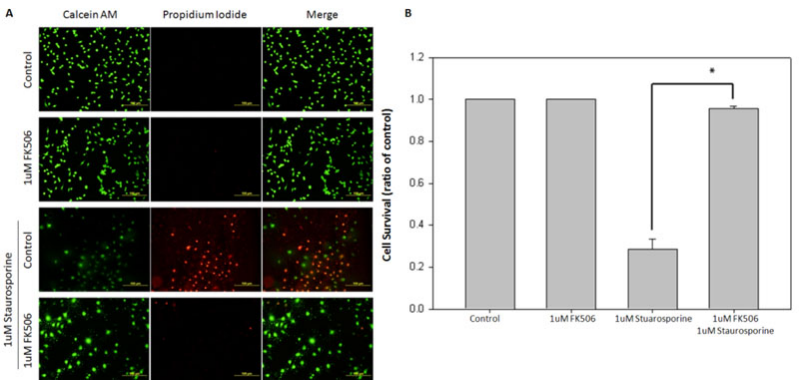
FK506 (tacrolimus) protects 661w cell cultures from staurosporine-induced cell death. **A**: FK506 (1 μM) significantly protected 661w neuronal cell cultures from staurosporine-induced apoptosis. Cell survival was monitored with the calcein-AM/propidium iodide cell-survival assay (scale bar=100 µm). The 661w neuronal cell cultures were treated with 1 μM FK506, with 1 μM staurosporine, with both 1 μM FK506 and 1 μM staurosporine, or with vehicle for 24 h. **B**: The 1 μM FK506 treatments significantly protected 661w neuronal cell cultures from 1 μM staurosporine-induced apoptosis (p<0.001, n=3). Error bars represent SEM.

FK506 is neuroprotective against several forms of toxicity as well as in several in vivo [5,6] and in vitro models [7,8]. Although FK506 is significantly neuroprotective outside of the eye, we want to determine whether FK506 protects ocular neuronal cell cultures from staurosporine-induced-apoptosis. The 661w neuronal cell cultures were processed for determination of apoptosis using calcein AM/propidium iodide double-staining [34] following 1 μM staurosporine and 1 μM FK506 treatments. Virtually all 661w neuronal cell cultures were alive when untreated or treated with 1 μM FK506. In contrast, when the 661w neuronal cell cultures were exposed to 1 μM staurosporine for 24 h, only 28±0.05% of the cell cultures survived. The addition of 1 μM FK506 significantly protected the 661w neuronal cell cultures (p≤0.001) from 1 μM staurosporine-induced apoptosis over the 24 h treatment, by increasing the survival rate to 95±0.01 (p=012; Figure 6).

### FKBP51 overexpression protected 661w cell culture from staurosporine induced caspase-3 activation

**Figure 7:**
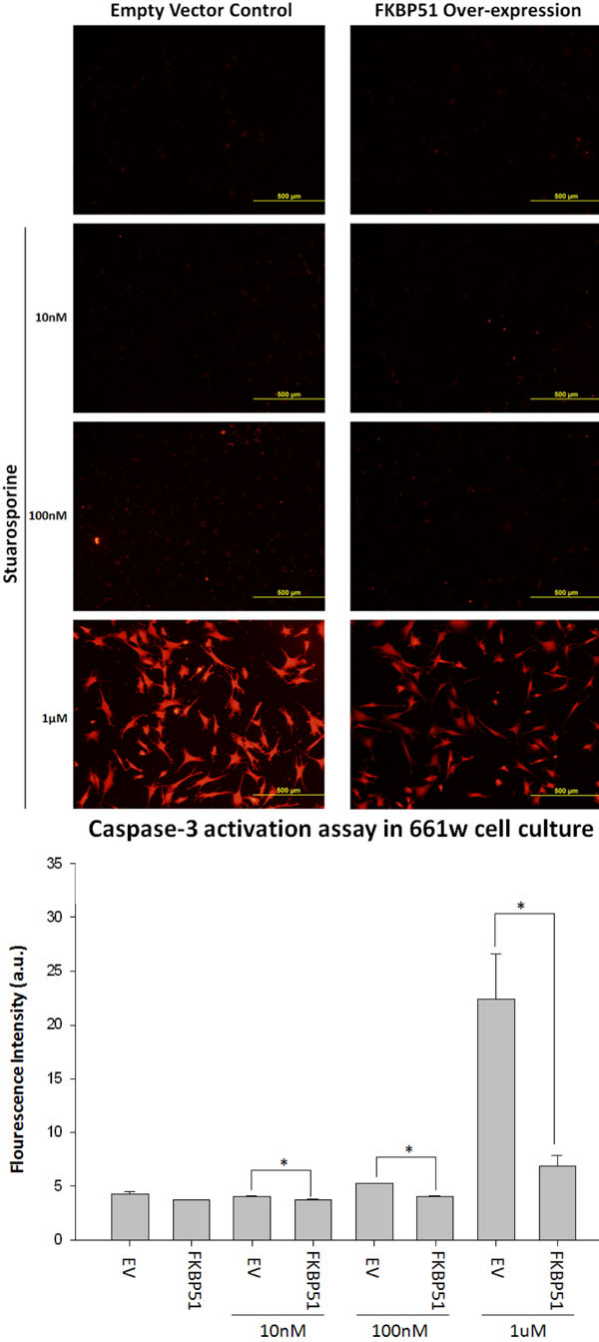
FK506 binding protein 51 overexpression protects 661w cell culture from staurosporine-induced caspase 3 activation. Staurosporine treatments for 6 h significantly increased caspase 3 activation, a marker of apoptosis, in 661w cell culture. FKBP51 overexpression in 661w cell cultures significantly decreased caspase 3 activation during staurosporine treatments. Caspase 3 activity was assayed using a colorimetric caspase 3 detection kit. Intensity was measured using Image J. FKBP51 significantly protected against caspase 3 activation during staurosporine treatment at 10 nM (p=0.006, n=3), 100 nM (p=0.001, n=3), and 1 μM (p<0.001). Significance was obtained through ANOVA (ANOVA) and the Mann–Whitney test. Error bars represent SEM.

Apoptosis accounts for most of the neuronal cell death observed during neurologic disorders [3]. It would be advantageous to prevent apoptosis to uniformly treat a wide range of neurologic disorders. Staurosporine was used to induce apoptosis [22-24]. We investigated whether an increase of FKBP51 protein protected against caspase-3 activation, a common marker of apoptosis. Six hours of staurosporine treatment was used to induce a significant amount of caspase-3 activation. FKBP51 overexpression significantly decreased the amount of caspase-3 activation at all three concentrations of staurosporine: 10 nM (p=0.006), 100 nM (p=0.001), and 1 μM (p<0.001; Figure 7).

## Discussion

FKBP51 has diverse physiologic functions. FKBP51 is a chaperone protein that aids in the transport of several hormones and hormone receptors. It is increased in several forms of cancer. It may also be increased in prostate cancer patients. This increase of FKBP51 is a suggested cause for positive feedback from androgen and the androgen receptors. This promotes cell survival and growth in these non-neuronal cells [35-37]. An increase in FKBP51 protein levels has been shown to cause resistance to chemotherapeutic agents that induce apoptosis in cancerous tumors [35,38]. Even though this is a disease state, in which homeostasis is out of balance, it would be advantageous to more thoroughly understand whether increases in FKBP51 activity can promote cell survival and growth. In neurons, FKBP51 promotes microtubule stability and elongation. FKBP51 works with Hsp90 to bind phosphorylated tau [39]. FKBP51 catalyzes the cis-trans isomerization of the peptidyl–prolyl bonds (the PPIase reaction), allowing tau to be recycled. With a mutant or defective FKBP51 molecule, the PPIase reaction will not occur, causing an accumulation of phosphorylated tau proteins, potentially leading to an Alzheimer disease-like state [39]. Furthermore, FKBP51 is involved in several cell-signaling pathways that promote cell survival and neuroregeneration [16,40]. FKBP51’s diverse physiologic functions, including its prosurvival properties, make it an important molecule to continue researching.

In this study, we have shown that FKBP51 neuroprotects 661w neuronal cell lines from the apoptosis-inducing agent, staurosporine. Even though FKBP51 has been well researched for its protective properties outside of the central nervous system, its protective properties within neurons need more research [16,17]. FKBP51’s neuroprotective efficacy and function in other in vitro and in vivo models of ocular neurodegeneration need to be tested to determine its potential for treating neurodegenerative diseases and neurotrauma. The downstream molecules that FKBP51 interacts with appear to be similar to those that interact with FK506. Furthermore, a drug that targets FKBP51 without inhibiting calcineurin through FK506 and causing systemic immunosuppression would be beneficial. A drug that is a potential candidate is GPI1046. GPI1046 is an FK506 analog, a non-immunosuppressive immunophilin ligand that appears to have the same neuroprotective properties [12]. GPI1046 displays neurosurvival and regenerative activities in vivo and in vitro [41,42]. Further investigation of GPI1046 is needed to determine its value not only as a neuroprotective molecule, but also as an aid to characterizing immunophilins such as FKBP51.

FKBP51 is an important coactivator of the NF-κB signaling pathway [34]. NF-κB has both detrimental and prosurvival effects in neurons. NF-κB activity still remains an important signaling molecule to investigate. Many argue that NF-κB has a large effect on prosurvival genes, supported by the finding that NF-κB knockouts are lethal during development [30,43,44]. This present study demonstrates that an increase of FKBP51 can increase protein levels and the activation of NF-κB’s major subunit, NF-κB p65. Interestingly, FK506’s effect on NF-κB in neurons is still controversial [45,46]. Nevertheless, 1 μM of FK506 caused a phosphorylation of NF-κB p65 in 661w neuronal cell cultures after 30 min. The mechanism of this activation is still not understood; however, it may occur through FKBP51. Additional testing is needed to determine whether FKBP51 is essential to FK506 phosphorylation of NF-κB p65.

In summary, we have shown that an increase of FKBP51 protein protects 661w neuronal cell cultures from the apoptosis-inducing agent staurosporine. Both FK506 and FKBP51 share similar downstream signaling molecules, suggesting that utilization of FKBP51 by FK506. FKBP51 has has diverse physiologic functions in promoting several prosurvival pathways. A potential therapeutic intervention is to increase the function of FKBP51, which could increase the stability and duration of several FKBP51 downstream molecules to maintain or even increase cell survival.
